# Graphene Quantum Dots Modified Upconversion Nanoparticles for Photodynamic Therapy

**DOI:** 10.3390/ijms232012558

**Published:** 2022-10-19

**Authors:** Yuting Li, Yufei Wang, Hong Shang, Jing Wu

**Affiliations:** School of Science, China University of Geosciences (Beijing), Beijing 100083, China

**Keywords:** photodynamic therapy, photosensitizer, upconversion nanoparticles, graphene quantum dots, reactive oxygen species

## Abstract

Photodynamic therapy (PDT), as a novel technique, has been extensively employed in cancer treatment by utilizing reactive oxygen species (ROS) to kill malignant cells. However, most photosensitizers (PSs) are short of ROS yield and affect the therapeutic effect of PDT. Thus, there is a substantial demand for the development of novel PSs for PDT to advance its clinical translation. In this study, we put forward a new strategy for PS synthesis via modifying graphene quantum dots (GQDs) on the surface of rare-earth elements doped upconversion nanoparticles (UCNPs) to produce UCNPs@GQDs with core-shell structure. This new type of PSs combined the merits of UCNPs and GQDs and produced ROS efficiently under near-infrared light excitation to trigger the PDT process. UCNPs@GQDs exhibited high biocompatibility and obvious concentration-dependent PDT efficiency, shedding light on nanomaterials-based PDT development.

## 1. Introduction

Photodynamic therapy (PDT) is an emerging cancer treatment method combining light, oxygen, and a photosensitizer (PS) to produce highly cytotoxic reactive oxygen species (ROS) that cause cancer cell death [1,2,3]. Two kinds of mechanisms (type I and II) are involved in the PDT process. In most cases, the type II mechanism needs to transform ground-state triplet oxygen (^3^O_2_) into highly reactive singlet oxygen (^1^O_2_) and is limited by the concentration of O_2_. Different from the type II route, the type I process takes place through either electron or hydrogen atom abstraction by exciting PSs from the substrate and performs well even under low O_2_ conditions [4,5]. Compared with conventional cancer therapy methods, PDT is superior in minimal side effects, low cumulative toxicity, precise targeting therapy with no damage to neighboring normal tissues, and long-term therapeutic effect [6]; however, inherent shortages of PDT impede its clinical translation, such as limited depth of light penetration, inefficient ROS generation, and lacking ideal PSs.

In recent years, nanoparticles (NPs) have been tried to be used as PSs toward solving the associated problems with PDT [7,8]. Upconversion nanoparticles (UCNPs) are a new type of optical nanomaterials composed of rare earth elements with low bandgap and featured with narrow emission bands and excellent photostability [9,10,11,12]. The upconversion luminescence of UCNPs provides an alternative way to overcome the existing drawbacks of PDT [13]. The near-infrared (NIR) excitation light of UCNPs is a transparent window in tissue and permits deep tissue penetration. Besides that, due to the adjustable doped ratio of rare earth elements, the intramolecular charge transfer can be realized via excited state absorption and energy transfer, which provides a promising pathway for electron transfer aiding ROS generation [14,15]. Meanwhile, versatile nanocomposites based on UCNPs can be designed due to their easy surface functionalization and show great potential for PDT applications.

In this work, UCNPs (NaYF_4_:Yb/Er) were modified with graphene quantum dots (GQDs) to obtain a composite (UCNPs@GQDs) which was used as PSs for PDT in the NIR therapeutic window. The surface of UCNPs was activated by the N-doped GQDs, which were synthesized by using folic acid (FA) as a carbon source. GQDs were selected as the modification agent due to their rich valence band could stabilize electrons/holes [16]. GQDs displayed some advantages which were beneficial for PDT, such as excellent photoluminescent features, corrosion resistance, high water solubility, high photo/pH-stability, in vitro and in vivo biocompatibility, and efficient ROS generation [17,18]. Here, the ROS generation performance was dramatically promoted in the composite of UCNPs@GQDs due to the up-convert energy transition and dexter excitation transfer (DET), which was identified by photoluminescence (PL) spectra. ROS production was accompanied by the chemical adsorption of oxygen, which was reduced by the photo-induced electrons to be O_2_^•−^ and then was oxidized to be ^1^O_2_ by holes. Significantly, upon protonation, the charge separation was further increased due to the improved light absorption capacity and limited electron–hole recombination. Combined with the excellent charge separation and promoted oxygen diffusion and ROS release, the modified UCNPs led to excellent PDT performance through the type I mechanism.

## 2. Results

### 2.1. Characterization of UCNPs@GQDs

**Figure 1:**
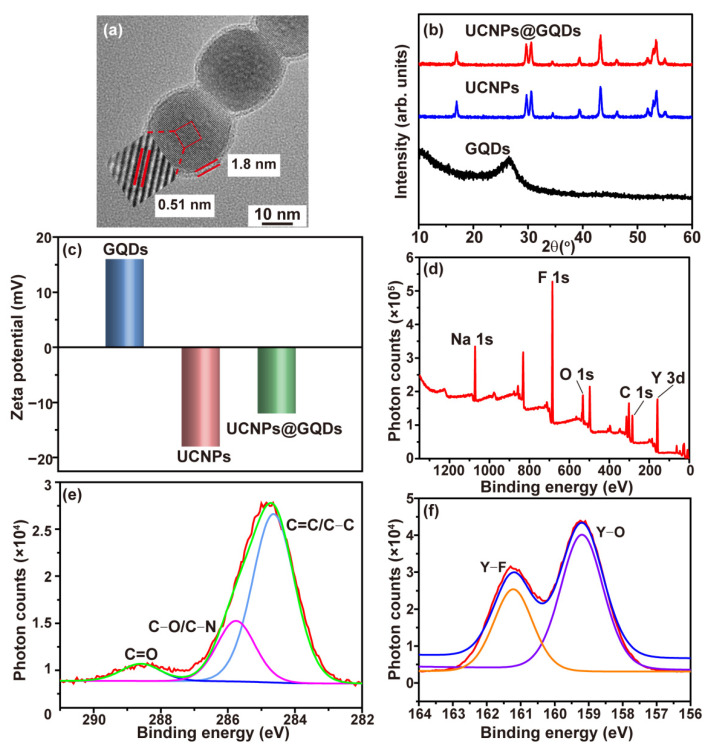
Characterization of UCNPs@GQDs. (**a**) HRTEM image of UCNPs@GQDs. The inset was the lattice fringes of UCNPs. (**b**) XRD pattern of UCNPs@GQDs. (**c**) Zeta potential of GQDs, UCNPs, and UCNPs@GQDs. (**d**) XPS survey spectrum of UCNPs@GQDs. (**e**) High-resolution C 1s XPS profiles of UCNPs@GQDs. (**f**) High-resolution Y 3d XPS profiles of UCNPs@GQDs.

The morphology of UCNPs@GQDs was examined by transmission electron microscopy (TEM) and scanning electron microscope (SEM) (Figure 1a and Figure S1b). UCNPs@GQDs exhibited a core-shell structure, and the thickness of the shell was about 1.8 nm. All of the nanoparticles displayed similar shapes and equal sizes. UCNPs lattice was observed, and the width of lattice fringes was 0.51 nm which was consistent with the characteristic packing of UCNPs demonstrating the core-shell structure of UCNPs@GQDs. Elemental mapping images in Figure S2 also proved this and showed that the distribution of elements C, N, and O was consistent with that of elements Y and Yb. The X-ray diffraction (XRD) patterns of the synthesized GQDs, UCNPs, and UCNPs@GQDs are presented in Figure 1b. XRD profile of UCNPs clearly revealed their crystallization of the α phase (JCPDS 77-2042) [19]. The obvious peak located at 25° in the XRD pattern of GQDs arose from the 002-lattice plane [20]. The XRD pattern of UCNPs@GQDs demonstrated their well-crystallized structure, which was the same as that of UCNPs. The zeta potential of GQDs, UCNPs, and UCNPs@GQDs was detected in pH = 7 aqueous medium and is shown in Figure 1c. It exhibited that the zeta potential of GQDs was opposite to that of UCNPs. That was why the two particles were mutually combined to form a composite. Meanwhile, the decrease in zeta potential for UCNPs@GQDs proved the successful loading of GQDs. The detailed bond structure on the UCNPs@GQDs surface was confirmed by X-ray photoelectron spectroscopy (XPS) spectra (Figure 1d–f). In the high-resolution XPS spectra of C 1s of UCNPs@GQDs, C=C (284.2 eV), C-O (284.9 eV), and C=O (288.4 eV) peaks were observed and demonstrated the existence of GQDs on the surface of UCNPs. The high-resolution XPS spectra of Y 3d also confirmed this result. In addition to the presence of the Y-F (161.2 eV) peak of NaYF_4_, Y-O (159.2 eV) peak also presented with higher strength, possibly because the oxygen-containing functional groups existing on the surface of GQDs filled within lattice defects of NaYF_4_. As a result, DET may occur between GQDs and UCNPs. The convergence of the XPS profiles of C 1s and Y 3d was good, demonstrating the credibility of these data (Figure S3).

**Figure 2:**
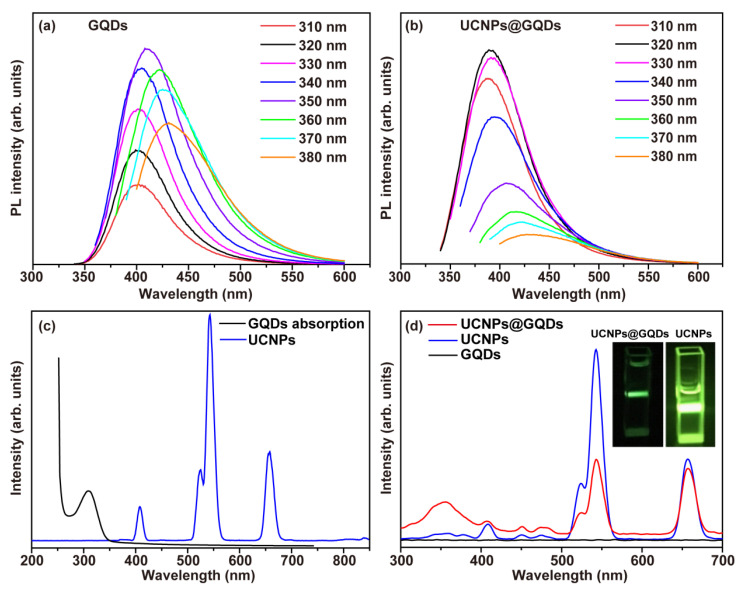
Optical properties of UCNPs@GQDs. (**a**) PL spectra of GQDs detecting with different excitation wavelengths. (**b**) PL spectra of UCNPs@GQDs detecting with different excitation wavelengths. (**c**) The spectral overlap of GQDs absorption and UCNPs emission (λ_ex_ = 980 nm). (**d**) Upconversion luminescence spectra of UCNPs@GQDs, UCNPs, and GQDs (λ_ex_ = 980 nm). The insert is the photograph of UCNPs@GQDs and UCNPs upon 980 nm laser irradiation.

As one kind of carbon dot, GQDs also have excitation wavelength-dependent PL emission [21]. GQDs emission exhibited a red shift as the excitation light shifted to a longer wavelength (Figure 2a). The strongest emission of GQDs was located at 412 nm when the excitation wavelength was 350 nm. Such properties were inherited in UCNPs@GQDs PL spectra, which also had the red-shift effect and gave the maximum emission at the wavelength of 382 nm excited by the light of 320 nm (Figure 2b). The PL emission of UCNPs@GQDs gives them the potential to be fluorescent imaging materials. The decrease in emission intensity of UCNPs@GQDs was referred to as the result of the blocking of n-π* transitions of oxygen-containing functional groups on the surface of GQDs. Based on XPS spectra, the oxygen-containing functional groups partially filled in the lattice defects of UCNPs, making some n-electrons participate in forming feedback bonds, which weakened the PL emission of UCNPs@GQDs. These results indirectly proved the successful formation of the core-shell structure in UCNPs@GQDs. Meanwhile, under the irradiation of 980 nm laser, the main emission peaks of the UCNPs located at 407, 525, 543, and 657 nm and were attributed to ^2^H_9/2_→^4^I_15/2_, ^2^H_11/2_→^4^I_15/2_, ^4^S_3/2_→^4^I_15/2_, ^4^F_9/2_→^4^I_15/2_ transitions of Er^3+^ ions, respectively. GQDs gave a broad UV-Vis absorption band which centered at ~310 nm but had no overlap with the emission of UCNPs, indicating there was no Förster resonance energy transfer between UCNPs and GQDs (Figure 2c) [22,23]. The emission spectra of UCNPs@GQDs, UCNPs, and GQDs were detected through exciting by a 980 nm laser to explore the possible energy transfer mechanism between the UCNPs and GQDs. Compared with the emission spectrum of UCNPs, the main emission peak located at 543 nm significantly dropped as well as a new emission peak appeared at 354 nm in the emission spectrum of UCNPs@GQDs (Figure 2d). The new emission at the short wavelength availed electron ionization in high-energy states to generate ROS in an aqueous environment. The emission intensity of UCNPs@GQDs was dramatically quenched by GQDs, giving a promise for cytotoxic ^1^O_2_ generation [24]. The inset photograph in Figure 2d also clearly showed that the upconversion emission of UCNPs was partially quenched by the incorporated GQDs. Pure GQDs gave no upconversion emission irradiated by the 980 nm laser. As a result, the synthesized UCNPs@GQDs can be used as a potential PDT agent activated by NIR lights.

### 2.2. In Vitro ROS Detection for UCNPs@GQDs

**Figure 3:**
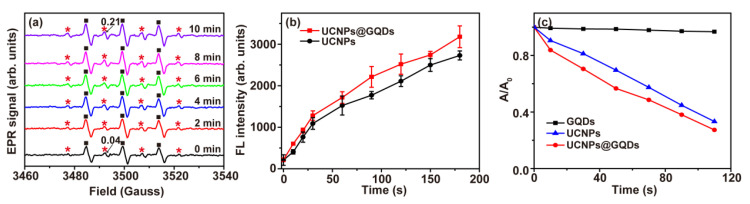
In vitro ^1^O_2_ detection for UCNPs@GQDs. (**a**) EPR spectra of UCNPs@GQDs were detected under the irradiation of a 980 nm laser for different times. DMPO was used as the specific scavenger agent to detect ·OH generation. The EPR signals marked with red asterisk symbols were assigned to DMPO-·OH. The EPR signals marked with black square symbols were assigned to ^1^O_2_. (**b**) FL intensity curves of SOSG were detected at 525 nm upon treatment by UCNPs and UCNPs@GQDs and subsequent 980 nm laser irradiation in different time duration. (**c**) Absorption decay curves of DPBF were detected at 420 nm upon treatment by UCNPs@GQDs and subsequent 980 nm laser irradiation in different time duration.

·OH and ^1^O_2_ are two kinds of ROS that are cytotoxic to cancer cells and can kill them by activating cell apoptosis or necrosis by attacking organic compounds encountered in the PDT process. Room-temperature electron paramagnetic resonance (EPR) spectra were collected to detect ·OH generated by UCNPs@GQDs under the irradiation of a 980 nm laser. DMPO was utilized as a specific detection reagent for ·OH to examine the variation of ·OH generation with the irradiation time. In Figure 3a, the characteristic 1:2:2:1 EPR signals (marked with red asterisk symbols) were assigned to DMPO-·OH and confirmed the generation of ·OH, which increased with the time of laser irradiation. In this system, DMPO was oxidized by ^1^O_2_ to be 5,5-dimethyl-2-oxopyrroline-1-oxyl (DMPOX), which gave triplet signals in EPR spectra (marked with black square symbols), indicating the existence of ^1^O_2_ in this system. Meanwhile, ^1^O_2_ generation was further detected by two specific fluorescent probes, Singlet Oxygen Sensor Green (SOSG) and 1,3-diphenylisobenzofuran (DPBF). SOSG was sensitive to ^1^O_2,_ while it did not react with ·OH or superoxide. SOSG reacted with ^1^O_2_ to give green FL, which became more intense with the increasing amount of ^1^O_2_. As shown in Figure 3b and Figure S4a,b, the FL intensity of SOSG rapidly increased with the presence of both UCNPs and UCNPs@GQDs under the 980 nm laser irradiation. Compared with the FL intensity curve of UCNPs, the slope of the curve of UCNPs@GQDs was larger, indicating more ^1^O_2_ generation induced by UCNPs@GQDs. Then, ^1^O_2_ production of UCNPs@GQDs was investigated by DPBF, which was a commercial fluorescent probe and irreversibly reacted with ^1^O_2_ to be 1,2-dibenzoylbenzene (DBB) (Figure S4c). The conjugated structure in DBB was broken and resulted in the decrease of absorbance of DPBF. Upon irradiation by a 980 nm laser, the absorbance intensities were decreased by UCNPs and UCNPs@GQDs, while GQDs brought no effect on the absorbance intensity (Figure 3c and Figure S3d). Both UCNPs and UCNPs@GQDs induced ^1^O_2_ generation. The irradiation time was longer, and the decrease in the absorbance intensity was faster (Figure S4e,f). The slope of the curves in Figure 3c represented the efficiency of ^1^O_2_ generation. It was obvious that the efficiency of ^1^O_2_ generation induced by UCNPs@GQDs was higher than that of UCNPs. All these results demonstrated the capability of UCNPs@GQDs for ROS generation, which is promising for their application in PDT.

### 2.3. PDT Assessment for UCNPs@GQDs

**Figure 4:**
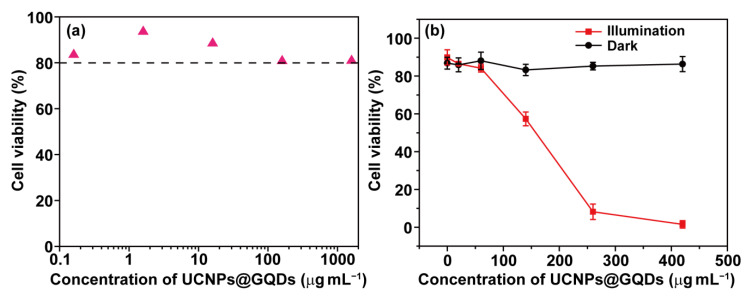
PDT assessment for UCNPs@GQDs. (**a**) Biocompatibility of UCNPs@GQDs was assessed without irradiation of a 980 nm laser. (**b**) Cell viability of HeLa cells treated by UCNPs@GQDs was determined by Live/Dead assay kit.

Before PDT assessment, the cytotoxicity of UCNPs@GQDs was evaluated by Live/Dead assay kit to make sure whether their biocompatibility was appropriate for PDT tests. A series of UCNPs@GQDs concentrations ranging from 0.16 to 1600 μg∙mL^−1^ were assessed in order to test all the concentrations used in PDT tests. Without NIR laser irradiation, the cells treated by UCNPs@GQDs exhibited high cell viability, which was above 80% at the concentration even up to 1600 μg∙mL^−1^ suggesting the good biocompatibility of the synthesized UCNPs@GQDs (Figure 4a and Figure S5a). UCNPs@GQDs-treated cells followed by NIR laser irradiation had an apparently declined cell viability compared with the dark group demonstrating UCNPs@GQDs had perfect PDT efficacy for tumor cells (Figure 4b and Figure S5b). The data in Figure 4b and Figure S4c also indicate that UCNPs@GQDs displayed poor toxicity to the cells under dark conditions.

**Figure 5:**
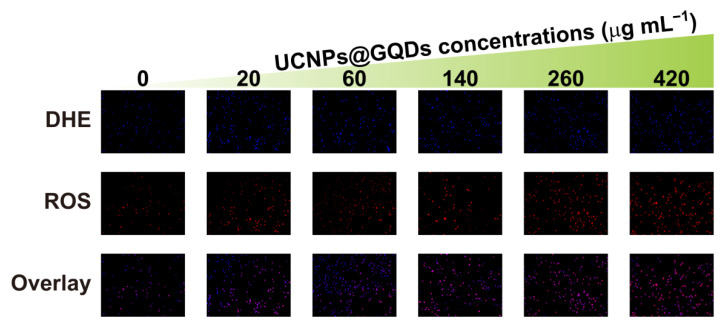
Intracellular ROS detection using DHE as the specific fluorescent probe. HeLa cells were treated with different concentrations of UCNPs@GQDs and illuminated by a 980 nm laser.

High ROS generation is indispensable for a PS and plays an important role in the PDT process. The amount of ROS is kept low in a normal intracellular microenvironment of cancer cells and easily neutralized by in vivo antioxidants. PS can break this balance by inducing a large amount of ROS generation and initiating cell apoptosis. Dihydroethidium (DHE) was used as a specific FL probe to detect intracellular ROS generation induced by UCNPs@GQDs under NIR laser irradiation. Intracellular DHE gave blue FL emission (λ_ex_ = 370 nm) before illumination and reacted with ROS to give red FL emission (λ_ex_ = 535 nm) after illumination. As shown in Figure 5, the red FL of ROS augmented with the increase in UCNPs@GQDs concentration, and the results were consistent with the data on cell viability.

## 3. Discussion

Here, GQDs-modified UCNPs were used as a novel PS in the PDT process induced by NIR light. The composite UCNPs@GQDs were facilely prepared and exhibited excellent biocompatibility. Cytotoxic ROS was efficiently generated upon NIR light irradiation. As a result, this work provides a novel paradigm with highly integrated functionalities, which exhibits excellent prospects not only for imaging-guided PDT but also encourages us to further explore new types of multifunctional NPs for biomedical applications.

## 4. Materials and Methods

### 4.1. Reagents and Materials

FA, oleic acid, 1-octadecene, and Tris-HCl buffer were all purchased from Sigma-Aldrich Chemical Co. Ltd. (St. Louis, MO, USA). Ethanol (99.7%) and sodium hydroxide were purchased from Sinopharm Chemical Reagent Co. Ltd. (Shanghai, China). Hydrochloric acid (HCl), methanol (MeOH), and cyclohexane were supplied by Beijing Chemical Reagent Co. Ltd. (Beijing, China). SOSG, DPBF, and DHE were purchased from Leyan reagent Co. Ltd. All chemical reagents were used as received without further purification.

### 4.2. Apparatus

High-resolution TEM (HRTEM) images were recorded by a JEOL-1400 transmission electron microscope (JEOL, Tokyo, Japan). The PL spectra were performed on an Agilent Cary Eclipse spectrofluorometer (F-7000, Hitachi, Japan). The silt width was set as 5 nm. The voltage of the photomultiplier tube (PMT) was controlled to be 700 V. UV-vis absorption spectra were collected on a Perki-nElmer Lambda 950 spectrophotometer. SEM images were taken on a field emission scanning electron microscope (FESEM, Carl Zeiss AG, Germany). XRD spectra were collected on Bruker D8 diffractometer with the Cu Kα radiation. XPS was measured by an X-ray photoelectron spectrometry (AXIS SUPRA, SHIMADZU, Japan). EPR spectra were measured on a Bruker E500 spectrometer.

### 4.3. Material Synthesis

#### 4.3.1. Synthesis of GQDs

GQDs were synthesized according to a previous method with some modifications [25]. Then, 10 mg FA was dissolved in 15 mL of deionized water. It was uniformly dispersed under ultrasonic conditions and then transferred into a 25 mL Teflon-lined stainless-steel autoclave. The FA solution was heated at 240 °C for 6 h. After cooling to room temperature, a dark brown liquid was obtained and centrifuged at 12,000 rpm for 10 min to remove the sediment. The product was freeze-dried and stored at room temperature for further use. The obtained N-doped GQDs were sphere-like NPs of about 2–3 nm size (Figure S1a) and consisting of nitrogen and functional groups of –OH, –NH_2,_ and –COOH.

#### 4.3.2. Synthesis of UCNPs

The coprecipitation method was adopted to synthesize the UCNPs (NaYF_4_:Yb/Er) following a recipe from the literature with some slight modifications [26]. Then, 3.2 mL Y(OAc)_3_ (0.2 M), 720 μL Yb(OAc)_3_ (0.2 M), 80 μL Er(OAc)_3_ (0.2 M), and 6 mL oleic acid were mixed well in a 100 mL two-neck flask, and the reaction was carried out at 150 °C for 30 min under vigorous stirring to evaporate the water. Then, 14 mL1-octadecene was added to the mixture, and the reaction was kept at 150 °C for another 30 min. After that, the temperature of the mixture was cooled to ~50 °C. Then, 4 mL NaOH solution (0.5 M, dissolved in MeOH) was mixed with 8 mL NH_4_F solution (0.4 M) and quickly added into the reaction system, which was kept at 50 °C for 30 min. Then, the temperature of the system was warmed up to 100 °C and held for 20 min to remove MeOH. After three cycles of pumping and charging with high-purity nitrogen, the reaction was carried out at 290 °C for 2 h under nitrogen protection. After the reaction was completed and cooled to room temperature, ethanol, cyclohexane, 2 M HCl:ethanol (V:V = 1:1), and deionized water were used to clean the reactant by centrifugation, and the solid was collected for subsequent use.

#### 4.3.3. Synthesis of UCNPs@GQDs

In total, 4 mL GQDs (1 mg·mL^−1^) were uniformly mixed with 12.8 mg UCNPs and then sonicated for 20 min. Subsequently, the mixture was loaded into a Teflon line stainless steel autoclave and reacted at 80 °C for 6 h. After the reaction, it was cooled to room temperature. The product was mixed with deionized water and centrifuged twice at 12,000 rpm for 15 min. The solid was collected and dispersed in 0.05 M Tris-HCl buffer (pH = 7.4) for further use.

### 4.4. Assessment of ^1^O_2_ Generation

SOSG was used as a ^1^O_2_ indicator to evaluate the ^1^O_2_ generation [27]. SOSG reacted with ^1^O_2_ to give bright green fluorescence (FL) (λ_ex_/λ_em_ = 504/525 nm). GQDs, UCNPs, or UCNPs@GQDs were suspended in 2 mL water at the concentration of 500 μg·mL^−1^ in the presence of 10 μM SOSG. The mixed suspension was exposed to laser irradiation (0.8 W·cm^−2^, 980 nm) for 0, 10, 20, 30, 60, 90, 120, and 180 s, and the FL at different time points was measured with a fluorimeter. Further, ^1^O_2_ generation was monitored by DPBF via UV-vis spectroscopy [28]. DPBF (100 μg·mL^−1^) was dispersed in GQDs, UCNPs, or UCNPs@GQDs solutions. Then, the mixed solution was irradiated by a 980 nm laser with the power of 0.8 W·cm^−2^. The change of absorption of DPBF at 412 nm was recorded over time.

### 4.5. PDT Tests

#### 4.5.1. Biocompatibility of UCNPs@GQDs

HeLa cells were seeded at a density of 1 × 10^4^ cells per well in a 96-well plate and cultured at 37 °C for 24 h in a 5% CO_2_ environment. After that, the culture medium was replaced by a series of UCNPs@GQDs solutions (dissolved in Tris-HCl) with concentration gradients (1.6 × 10^−4^, 1.6 × 10^−3^, 1.6 × 10^−2^, 0.16, and 1.6 mg·mL^−1^) and the cells continued to be cultured for 24 h. Afterwards, cell apoptosis was analyzed by staining cells with a Live/Dead assay kit (Calcein-AM/EthD-1, Invitrogen, CA, USA). The stained cells were observed by an FL confocal microscope, and the data were analyzed by the program Image-Pro Plus 6.0. Three independent experiments were carried out with the same concentration gradients of UCNPs@GQDs solutions.

#### 4.5.2. Cell Apoptosis Assays

HeLa cells were seeded at a density of 1 × 10^4^ cells per well in a 96-well plate and cultured at 37 °C for 24 h in a 5% CO_2_ environment. The 96-well plate is numbered from top to bottom and from left to right. The No. 1–3 rows on the 96-well plate were used for PDT tests, and the No. 4–6 rows were used as the control groups without NIR laser irradiation. After 24 h cell culture, UCNPs@GQDs solutions were infused into the wells in rows 1–6. The wells in rows No. 7 and 8 were infused with blank Tris-HCl buffer solution. Cells were incubated with UCNPs@GQDs for 6 h. After that, a 980 nm laser beam with the power of 0.8 W/cm^2^ was used to irradiate the cells in No. 1–3 and 7 rows for 20 min, and the irradiation was stopped for 1 min every 1 min. After another 48-h incubation, the cells were stained by a Live/Dead assay kit and observed by an FL confocal microscope. For intercellular ROS detection, the cells were stained by DHE and observed by an FL confocal microscope. The data were analyzed by the program Image-Pro Plus 6.0.

## Supplementary Materials

The following supporting information can be downloaded at: https://www.mdpi.com/article/10.3390/ijms232012558/s1.
