# Supplementary material for: Graphene Quantum Dots Modified Upconversion Nanoparticles for Photodynamic Therapy
Source: Int J Mol Sci. 2022 Oct 19;23(20):12558. doi: 10.3390/ijms232012558 (PMC9604409; doi:10.3390/ijms232012558)
Supplement: Supplementary file 1 [file ijms-23-12558-s001.zip › ijms-1978403-supplementary.pdf]

Supplementary Information

Graphene quantum dots modified upconversion nanoparticles for photodynamic therapy

Yuting Li,[#] Yufei Wang,[#] Hong Shang*, Jing Wu*

School of Science, China University of Geosciences (Beijing), Beijing 100083, China

*Corresponding authors: Hong Shang (Email: shanghong@cugb.edu.cn, TEL: +86-10-82322758); Jing Wu (Email: wujing@cugb.edu.cn, TEL: +86-10-82322758)

1. Characterization of UCNPs@GQDs

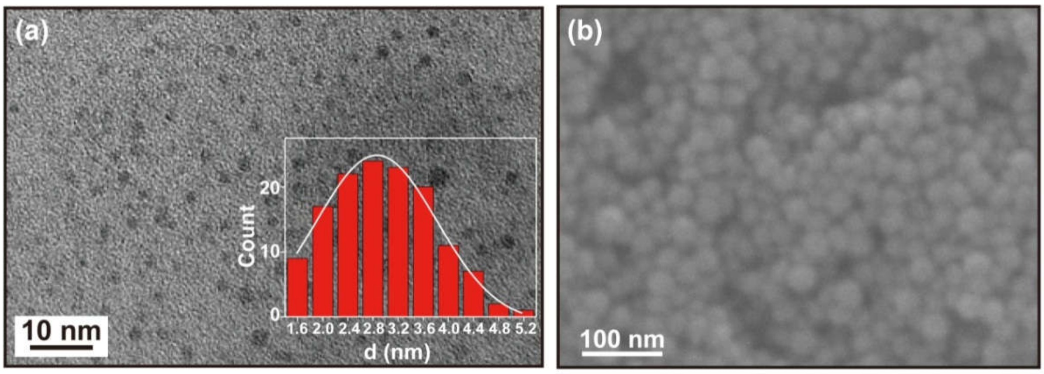

Figure S1. Characterization of GQDs and UCNPs@GQDs. (a) TEM image and size distribution of GQDs. (b) SEM image of UCNPs@GQDs.

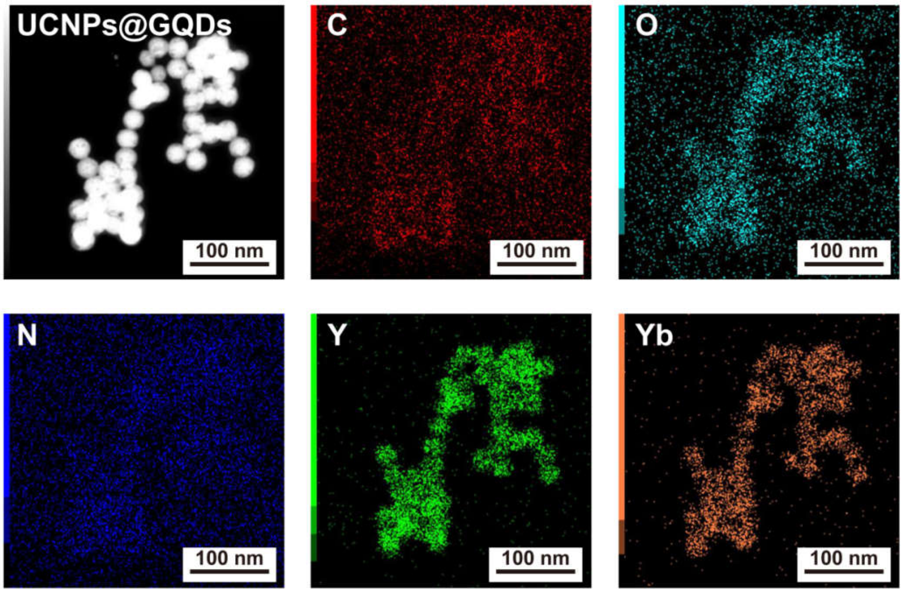

Figure S2. Elemental mapping of UCNPs@GQDs.

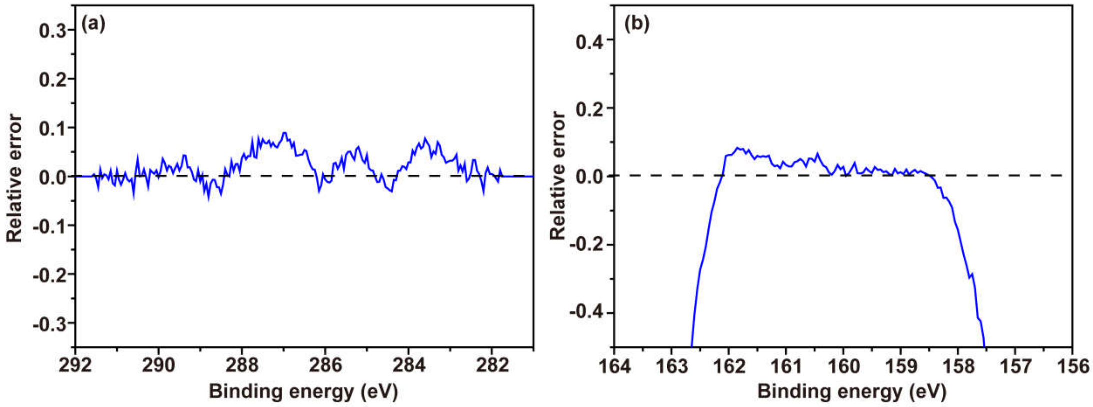

Figure S3. The convergence of XPS profiles. (a) The convergence of C1s XPS profiles of UCNPs@GQDs. (b) The convergence of Y 3d XPS profiles of UCNPs@GQDs.

2. $^1O_2$ detection

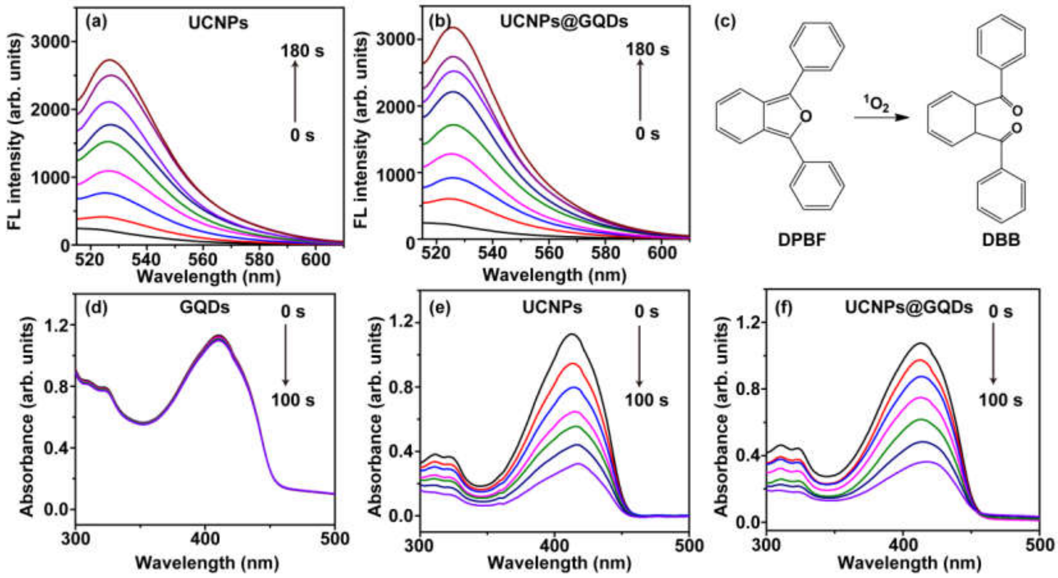

Figure S4. $^1O_2$ detection by SOSG and DPBF. (a and b) FL intensity curves of SOSG were detected at 525 nm upon treating by UCNPs and UCNPs@GQDs and subsequent 980 nm laser irradiation in different time duration. (c) The reaction between DPBF and $^1O_2$. (d-f) Absorption decay curves of DPBF were detected at 420 nm upon treating by UCNPs@GQDs and subsequent 980 nm laser irradiation in different time duration.

3. PDT assessment

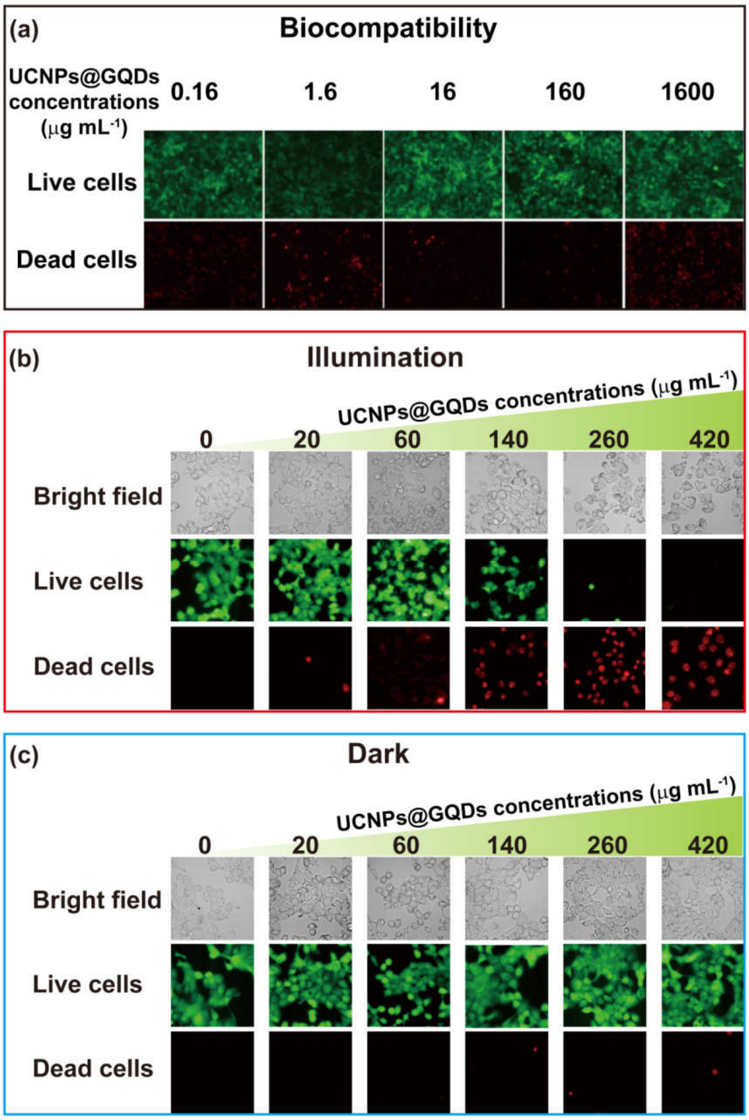

Figure S5. PDT assessment for UCNPs@GQDs. (a) Fluorescent images of HeLa cells treated by live/dead assay kit were taken after UCNPs@GQDs treatment without 980 nm laser irradiation in order to assess the biocompatible of UCNPs@GQDs. (b) Fluorescent images of HeLa cells treated by live/dead assay kit were taken after UCNPs@GQDs treatment with 980 nm laser irradiation in order to assess the PDT effect of UCNPs@GQDs. (c) Fluorescent images of HeLa cells treated by live/dead assay kit were taken after UCNPs@GQDs treatment without 980 nm laser irradiation for comparison.
